# Haematological manifestations, mechanisms of thrombosis and anti-coagulation in COVID-19 disease: A review

**DOI:** 10.1016/j.amsu.2020.06.035

**Published:** 2020-06-30

**Authors:** Ganesh Kasinathan, Jameela Sathar

**Affiliations:** Department of Haematology, Ampang Hospital, Jalan Mewah Utara, Pandan Mewah, 68000, Ampang, Selangor, Malaysia

**Keywords:** Coronavirus-19 disease, Pulmonary intravascular coagulopathy, Thromboembolism, Bleeding episodes

## Abstract

Coronavirus-19 disease (COVID-19), a zoonosis, was first reported in the city of Wuhan, province of Hubei, China in December 2019. The disease is caused by the Severe Acute Respiratory Syndrome-CoronaVirus-2 (SARS-CoV-2). As of 12^th^ of May 2020, 4,256,022 confirmed cases affecting 212 countries with 287,332 deaths have been reported. The common symptoms reported in patients with COVID-19 are fever, dry cough, dyspnoea and gastrointestinal symptoms such as vomiting and diarrhoea. Non-survivors often succumb due to widespread pulmonary intravascular coagulopathy, arterial and venous thromboembolism, disseminated intravascular coagulopathy (DIC), secondary hemophagocytic lymphohistiocytosis (sHLH), and multiorgan dysfunctional syndrome (MODS). All hospitalised patients should be monitored closely for thrombotic events. Patients who develop bleeding episodes should be managed according to standard DIC guidelines. The main objectives of this review are 1) to provide a succinct background of this novel disease 2) discuss the haematological presentations and mechanisms of thrombosis 3) emphasize the role of anti-coagulation prophylaxis 4) explore the management of coagulopathy 5) provide insight on management of patients with COVID-19 disease and pre-existing bleeding disorders.

## Highlights

• COVID-19 disease non-survivors often succumb to multiorgan dysfunctional syndrome.
• All hospitalised patients with COVID-19 disease are monitored closely for thrombotic events.
• Patients with COVID-19 disease who develop bleeding episodes are managed according to standard DIC guidelines.

## 1 Introduction: epidemiology, clinical presentation and diagnostic modalities

Coronavirus-19 disease (COVID-19), a zoonosis, was first reported in the city of Wuhan, province of Hubei, China in December 2019. The disease is believed to have originated from the Huanan human seafood wholesale market in Wuhan and is caused by the Severe Acute Respiratory Syndrome-CoronaVirus-2 (SARS-CoV-2) [1]. Bats and birds are known to be the main viral reservoirs with a well-documented history of animal-to-animal-to-human transmission, thus giving rise to the term zoonotic disease [2]. COVID-19 was declared a pandemic on the 11^th^ of March 2020 by the World Health Organisation (WHO) when 118,000 confirmed cases were reported, involving 114 countries worldwide with an approximate 4291 deaths. As of 12^th^ of May 2020, 4,256,022 confirmed cases affecting 212 countries with 287,332 deaths have been reported. The SARS-CoV-2 infection may manifest as mild, moderate, or severe on clinical presentation. The common manifestations reported in patients with COVID-19 are fever (most common symptom), dry cough, dyspnoea, sputum production, fatigue, myalgia, arthralgia, sore throat, headache and gastrointestinal symptoms such as nausea, vomiting and diarrhoea. Cytokine storm in the form of acute respiratory distress syndrome (ARDS), respiratory failure, sepsis and septic shock are among the severe presentations described.

This pandemic is attributed to human-to-human transmission (within 1 m) caused by respiratory droplets such as coughing and sneezing, and by indirect contact with contaminated surfaces or objects including thermometers and stethoscopes [3]. Subsequent studies have also shown that the virus is found in saliva, faeces, urine and semen [4].

This novel virus belongs to the family of beta coronaviruses, a positive-sense single stranded RNA with a lipid bilayer envelope [5]. Whole genome sequencing of the virus has revealed similar homology to other coronaviruses which have been implicated in respiratory outbreaks such as the Severe Acute Respiratory Syndrome (SARS) in 2002–2003 and Middle East Respiratory Syndrome (MERS) in 2012–2013 [6].

There are two main tests employed in the diagnosis of COVID-19; the first, is a molecular-based method comprising of real-time Reverse-Transcriptase Polymerase Chain Reaction (RT-PCR) of samples obtained from a nasopharyngeal/oropharyngeal swab or sputum and the second, is a serological immunoassay (combined IgM/IgG) analysis of the peripheral blood [7]. The viral load is often low during the early phase of the disease (Day 1–4) and also in patients who are recovering from the infection but they may still be infectious [8]. This low virus yield leads to false negative results on the RT-PCR. Another reason for a similar false negative result is a sample taken which is stored too long (extended storage) before being processed which results in degeneration of the virus RNA. On the other hand, sample contamination with surfaces containing high viral load such as gloves or contact with secretions from other true positive patients may produce false positive results. Serology-based testing (SBT) is not useful to diagnose new active infection but identifies patients who have been infected with COVID-19 infection at least 1–3 weeks previously as it is designed to detect antibodies produced in response to SARS-CoV-2 [9]. Since almost 30% of adults and a large number of children infected by COVID-19 are asymptomatic, a simple finger-prick SBT would be a cost-effective option to fulfil the needs for mass or population based screening [9].

At this time of writing, there is no readily available evidence from reliable randomised controlled trials (RCTs) on any potential therapy or prophylaxis which may provide clinical benefit in patients with COVID-19 infection. There are many on-going active clinical trials across the globe. Repurposed agents with most clinical evidence that have demonstrated in-vitro activity against SARS-CoV-2 are anti-malarial drugs (hydroxychloroquine or chloroquine) and anti-retroviral drugs (lopinavir/ritonavir) [10]. However, clinical trials have not confirmed clear efficacy with any of these drugs. It is also interesting to note that investigational convalescent plasma collected from individuals who have recovered from COVID-19 may be promising as a treatment [10]. The efficacy and safety of this treatment is currently being investigated in clinical trials.

Non-survivors often succumb to COVID-19 infection due to widespread pulmonary intravascular coagulopathy-pulmonary microvascular thrombosis and hemorrhage (PIC), arterial and venous thromboembolism including cerebral infarcts, disseminated intravascular coagulopathy (DIC), secondary hemophagocytic lymphohistiocytosis (sHLH), acute kidney injury and multiorgan dysfunctional syndrome (MODS).

## 2 Hematological manifestations

There are various hematological changes encountered in a patient with COVID-19 disease. Table 1 illustrates the various studies and findings of important hematological parameters in COVID-19 disease.

**Table 1:** Studies and findings of important hematological parameters in COVID-19 disease.

Author (year)	Country	Study period	Sample size	Hematology parameter studied	Study findings
Huang (2020)	Jinyintan Hospital, Wuhan, China	16th December 2019-2nd January 2020	41	Lymphopenia (lymphocyte count <1.0 x 10^9^/L)	85% of patients requiring intensive care demonstrated lymphopenia vs. 54% of patients that did not require intensive care (p = 0.045)
Wang (2020)	Zhongnan Hospital, Wuhan, China	1st January- 3^rd^ February 2020	138	Lymphocyte count as a continuous variable, x10^9^/L	ICU cases presented with lower lymphopenia (median:0.8, IQR: 0.5–0.9) vs. non-ICU cases (median: 0.9, IQR: 0.6–1.2); p = 0.03. Longitudinal decrease was noted in non-survivors.
Wu (2020)	Jinyintan Hospital, Wuhan, China	25th December 2019–13th February 2020	201	Lymphocyte count as a continuous variable, x10^9^/mL in a bivariate Cox regression model	Lower lymphopenia was associated with ARDS, (p < 0.001 in the incremental model); the association with survival did not reach significance, p = 0.11
Fan (2020)	National Centre for Infectious Diseases, Singapore	23rd January- 28^th^ February 2020	69	Thrombocytopenia; platelet count <100 × 10^9^/L.	Thrombocytopenia was not associated with ICU care either at admission (p = 0.67) or as a nadir during hospital stay (p = 0.69)
Yang (2020)	Jinyintan Hospital, Wuhan, China	24th December 2019-9th February 2020	52	Platelet count treated as a continuous variable ( × 10^9^/L)	Platelet count in non-survivors was 191 (63) and 164 (74) in survivors
Wu (2020)	Jinyintan Hospital, Wuhan, China	25th December 2019–13th February 2020	201	hs-CRP >5 vs. ≤5 mg/L in a bivariate Cox regression model	Higher hs-CRP was associated with ARDS, p = 0.008
Wu (2020)	Jinyintan Hospital, Wuhan, China	25th December 2019–13th February 2020	201	Serum ferritin >300 vs. ≤300 ng/mL in a bivariate Cox regression model	Hyperferritinemia was associated with ARDS (p = 0.003); the trend of an association with survival did not reach significance (p = 0.10)
Wang (2020)	Zhongnan Hospital, Wuhan, China	1st January- 3^rd^ February 2020	138	Elevated procalcitonin ≥0.05 ng/ml	75% of ICU patients presented with high procalcitonin vs. 21.6% of non-ICU patients, p < 0.001
Huang (2020)	Jin Yintan Hospital, Wuhan, China	16th December 2019-2nd January 2020	41	D-dimer as a continuous variable, in mg/L	Patients requiring ICU care demonstrated higher D-dimer levels, p = 0.0042

Leukopenia or normal total white cell count, lymphopenia (defined as absolute lymphocyte count <1.0 x 10^9^/L) and mild to moderate thrombocytopenia (platelet count > 50 x 10^9^/L) are commonly seen [11]. Lymphopenia and thrombocytopenia appear to predict disease severity for COVID-19. A defective immune response to the virus is believed to result in lymphopenia. SARS-CoV-2 is thought to inhibit bone marrow hematopoiesis through certain receptors resulting in lymphopenia and thrombocytopenia [12]. Other mechanisms for thrombocytopenia could be explained by the stimulation of anti-platelet autoantibodies by SARS-Cov-2 which triggers immune-mediated platelet destruction. The antibodies and immune complexes deposited on the surfaces of platelets predispose the platelets to easy destruction by the reticuloendothelial system [12]. It is also interesting to note that the presence of relative lymphocytosis is often associated with a milder disease and faster onset of spontaneous recovery. These features were demonstrated in a study of 51 COVID-19 patients in a centre in India who demonstrated a mean lymphocyte percentage of 40.6% with all patients requiring only symptomatic treatment [13].

Coagulopathic features which are associated with a more severe outcome in COVID-19 infection are prolongation of prothrombin time (PT) and activated partial thromboplastin time (aPTT), elevations of D-dimer, fibrinogen and fibrin degradation products (FDP) and decreased levels of antithrombin III [14]. It is recommended that every patient diagnosed with COVID-19 infection should have a baseline PT/aPTT, D-dimer and platelet count [14]. The aPTT may be less prolonged in COVID-19 infection as compared to typical sepsis induced disseminated intravascular coagulopathy (DIC) due to the increased levels of Factor VIII [14]. Von Willebrand factor which is also an acute phase reactant is quantitatively increased. A study consisting of 216 patients with COVID-19 infection showed 91% of the patients who had a prolonged aPTT was positive for lupus anticoagulant (LA) [15]. The aPTT was prolonged despite significant elevations in Factor VIII. The presence of LA is frequently associated with Factor XII deficiency which may occur secondary to the presence of factor XII inhibitors, which is also another cause for prolongation of aPTT [15]. Interestingly, both the presence of LA and Factor XII deficiency are not associated with bleeding tendency. Moreover, factor XII is not required for hemostasis. The role of LA in the thrombotic pathogenesis of the disease is unclear unlike the well documented thrombotic tendencies seen in the setting of antiphospholipid syndrome [16].

As the disease progresses, fibrinogen levels may fall resulting in a hypocoagulable phenotypic state thereby, increasing the risk of bleeding. Overt DIC (International Society on Thrombosis and Haemostasis-ISTH diagnostic score of 5 and higher) is seen more frequently in non-survivors [17]. It is also interesting to note that a four-fold increase in D-dimer predicts mortality in COVID-19 infection due to the increased risk of venous thrombosis and cytokine storm [18]. D-dimer is defined as a synthetic protein of the fibrin degradation product which contains two D fragments of the fibrin protein. Global coagulation assays including viscoelastic rotational thromboelastometry/thromboelastography (ROTEM/TEG) and thrombin generation assay (TGA) have been used to provide information on coagulation and fibrinolysis but the use to guide clinicians on anticoagulation therapy is still under investigation.

Other biochemical markers associated with a more severe presentation are elevations in lactate dehydrogenase (LDH), ferritin, C-reactive protein (CRP), procalcitonin, Troponin T, creatinine and liver enzymes. LDH is produced by almost all human cells and its increased level denotes damage to any cell type which expresses LDH. C-reactive protein is an acute phase reactant produced by the liver in response to inflammation.

## 3 Mechanisms of thrombosis and cytokine storm

There are several mechanisms which may explain hypercoagulability in COVID-19. Endothelial cells are known to play an important role in regulating haemostasis, fibrinolysis and vessel wall integrity. Endothelial cell injury activates a multitude of pro-inflammatory cytokines such as interleukin (IL)-1, IL-6, and tumour necrosis factor-alpha (TNF-alpha) [19]. This increase in inflammatory activity contributes to microvascular thrombosis including plugging of the pulmonary microvasculature and incidence of pulmonary embolism. The upregulation of tissue factor (TF) which is coupled with activated Factor V11 activity (TF-Factor VIIa complex) of the extrinsic coagulation pathway is associated with thrombin generation and fibrin deposition in various organs including the bronchoalveolar system [20]. On the other hand, hyperactive fibrinolysis leads to increased plasma concentrations of plasminogen and plasmin [21]. Increased plasmin cleavage activity could explain extreme elevations in D-dimers which correlates linearly with disease severity [21]. These mechanisms could explain the pathological findings of pulmonary tissue seen at autopsy which frequently reveal pulmonary intravascular coagulopathy and pulmonary embolism.

A subgroup of patients with COVID-19 disease may experience a cytokine storm which is characterised by fatal hypercytokinemia and often lead to multi-organ dysfunctional syndrome. Severe lymphopenia occurs in critically ill patients which could be explained by lymph node destruction, suppression of lymphocytes during lactic acidosis and binding of SARS-CoV-2 to angiotensin-converting enzyme 2 receptors on lymphocytes [22]. Serum ferritin levels were found to be significantly associated with disease severity and outcome. These patients demonstrate activation of T-helper-1 (Th1) function due to the increased concentration of inflammatory mediators such as IL-1B, IL-6, IL-12, IL-18, IL-33, CXCL10, CCL2 and TNF-alpha [22]. However, IL-6 appears to play a significant role through two signalling pathways: *cis* and *trans*. In *cis* signalling, IL-6 binds to the membrane-bound IL-6 receptor and gp130 which downstream Janus kinases and signal transducer and activator of transcription 3 proteins (JAK-STAT3) [23]. In *trans* signalling, the IL-6 binds to the soluble IL-6 which enhances secretion of vascular endothelial growth factor (VEGF) and IL-8 but reduces E-cadherin expression on endothelial cells [23]. Acute respiratory distress syndrome (ARDS) is the ultimate endpoint of a cytokine storm. A combination of numerous immune-active molecules including pro-inflammatory cytokines as mentioned above, chemokines, interleukins, colony stimulating factors and interferons contribute actively to the cytokine storm and ARDS.

## 4 Anti-coagulation therapy

As COVID-19 infection is interestingly associated with arterial and venous thrombosis, all hospitalised patients without evidence of active bleeding should be given prophylactic anticoagulation [24]. Mechanical thromboprophylaxis such as intermittent pneumatic devices (IPD) should only be used if pharmacological anticoagulation is contraindicated. The use of both concomitant pharmacological and mechanical anticoagulation should be avoided [24]. Prolonged PT and aPTT without evidence of bleeding should not preclude the use of anticoagulation. Patients who are on extracorporeal membrane oxygenation (ECMO) and/or continuous renal replacement therapy (CRRT) are believed have a higher risk of thromboembolism due to the increased inflammatory process [25]. The anticoagulation of choice is low molecular weight heparin (LMWH), unfractionated heparin (UFH) or subcutaneous fondaprinux [26]. Unfractionated heparin is a naturally occurring glycosaminoglycan with anti-thrombin and anti-inflammatory activity which has little interaction with drugs used to treat COVID-19 infection [27]. However, its usage as thromboprophylaxis is often not feasible in this setting due to the requirement for frequent plasma aPTT monitoring. Its short half-life favours the use in patients with high bleeding risk or renal impairment. Low molecular weight heparin such as enoxaparin 0.5 mg/kg once daily dosing is a better choice as they do not require frequent blood sampling [28]. However, in patients with severe renal impairment (creatinine clearance < 30 ml/min/1.73 m^2^), LMWH requires a well-callibrated anti-Factor Xa assay monitoring to ensure efficacy and to avoid drug toxicity [29]. Subcutaneous fondaparinux is contraindicated in advanced renal failure. Some authors advocate the use of therapeutic or intermediate-intensity dose anticoagulation such as subcutaneous enoxaparin 0.5 mg/kg twice daily as prophylaxis for patients without evidence of thrombosis, in particular, those being treated at the ICU with high D-dimers, fibrinogen and Factor VIII [30].

Patients with atrial fibrillation, prosthetic cardiac valves and pre-existing venous thrombosis who are currently treated with vitamin-K antagonist (warfarin) or direct oral anticoagulant (DOAC), it is important to note that these drugs may interfere with antiviral therapy used in COVID-19. In such a setting, an individual patient-based approach would be appopriate and a decision may be made to change the patient's existing treatment to a more convenient parenteral LMWH during the critical illness period [31].

The American Society of Hematology COVID-19 task force recommends that prophylactic anticoagulation should only be withheld in the presence of active bleeding or at a platelet count of less than 25 x 10^9^/L. Meanwhile patients with AF, mechanical cardiac valves and pre-existing thrombotic events should continue their full dose anticoagulation and are only advised to withhold such treatment at a platelet count of less than 30 x 10^9^/L. In addition, some authors have suggested the use of recombinant tissue plasminogen activator (r-tPA) in severely hypoxaemic patients not responding to therapeutic dose anticoagulation as pulmonary vasculature microthrombi and pulmonary embolism are often the causative factors [32].

Patients who are at high risk of developing thrombosis (Padua prediction score for risk of developing venous thromboembolism in hospitalised patients) such as; over 70 years of age, poor mobility, intensive care unit (ICU) admission, body mass index (BMI > 30 kg/m^2^) and history of active cancer should be given extended prophylactic anticoagulation upon discharge for a duration of 35–42 days [33]. Direct oral anticoagulant (DOAC) such betrixaban 60 mg daily or rivaroxaban 10 mg daily would be the anticoagulants of choice. On the other hand, those who are low risk may receive only low dose aspirin prophylaxis (aspirin 81 mg twice daily) for not less than 4 weeks in duration from discharge [33].

## 5 Management of coagulopathy

Coagulopathy without active bleeding should not warrant any transfusion of blood products as injudicious transfusion may lead to respiratory compromise and adverse events [34]. A patient with COVID-19 disease with bleeding episodes secondary to DIC should be treated like any other sepsis-induced DIC. They should receive red blood cells, platelet concentrates, cryoprecipitate (1 unit for every 10 kg per body weight) and virally inactivated plasma to maintain a platelet count of more than 50 x 10^9^/L and a plasma fibrinogen level of 1.5 g/L^34^. Antifibrinolytics such as tranexamic acid should be avoided in such situations because the excess fibrin need to be broken down. 4-factor prothrombin complex concentrate (4F-PCC) and fibrinogen concentrates would be the products of choice in coagulopathy associated with liver failure [35].

## 6 Bleeding disorders and COVID-19

World Federation of Hemophilia (WFH) Task Force recommends patients with bleeding disorders including severe hemophilia and von Willebrand disease hospitalised for COVID-19 infection receive higher than their usual doses of prophylactic factor replacement therapy to achieve adequate trough levels [36]. They should also receive concomitant prophylactic anticoagulation throughout their hospital admission. Those who are on non-factor replacement prophylaxis such as emicizumab, anti-tissue factor pathway inhibitor (anti-TFPI) and fitusiran, should continue their existing therapy throughout the illness [36]. The WFH task force has cautioned against the use of activated prothrombin complex concentrate (aPCC) in actively bleeding patients who are on emicizumab as this combination increases the risk of thrombotic microangiopathy [36].

## 7 Concluding remarks

In summary, the COVID-19 pandemic has inadvertently posed numerous challenges to our current medical knowledge, healthcare delivery and laboratory systems across the world. This review has emphasized on the various mechanisms of thrombosis, hematological aspects of the disease and imperativeness of anti-coagulation prophylaxis in COVID-19 patients including those with pre-existing bleeding disorders. Researchers are desperately finding definitive treatments for this disease to improve survival outcomes. All hospitalised patients should be monitored closely for thrombotic events. Patients who develop bleeding episodes should be managed according to standard DIC guidelines.

## Ethical approval

Ethical approval is not required as this is not a clinical trial.

## Funding

Self-funding.

## Author contributions

G.K. analysed the data, designed the paper, and contributed to the writing of the manuscript. J.S. made critical revisions and approved the final manuscript.

## Registration of research studies

1. Name of the registry: Not applicable.
2. Unique Identifying number or registration ID:
3. Hyperlink to your specific registration (must be publicly accessible and will be checked):

## Guarantor

Ganesh Kasinathan is the guarantor of this manuscript.

## Provenance and peer review

Not commissioned, externally reviewed.

## Declaration of competing interest

Both authors declare no potential conflicts of interests.
